# Zika convalescent macaques display delayed induction of anamnestic cross-neutralizing antibody responses after dengue infection

**DOI:** 10.1038/s41426-018-0132-z

**Published:** 2018-07-13

**Authors:** William G. Valiant, Yan-Jang S. Huang, Dana L. Vanlandingham, Stephen Higgs, Mark G. Lewis, Joseph J. Mattapallil

**Affiliations:** 1 F. Edward Hébert School of Medicine, Uniformed Services University, Bethesda, MD USA; 2 Biosecurity Research Institute, Department of Diagnostic Medicine/Pathobiology, College of Veterinary Medicine, Kansas State University, Manhattan, Kansas USA; 3 Bioqual, Rockville, MD USA

## Abstract

Structural similarities between Zika (ZIKV) and dengue virus (DENV) leads to the induction of cross-reactive responses. We have previously demonstrated that ZIKV exposed macaques significantly enhance DENV viremia. Here we show that this enhancement of DENV infection occurred in the presence of high levels of DENV cross-reactive IgG1 subclass of binding antibodies (bAb) with low DENV neutralizing antibody (nAb) activity (<1:10). The DENV-2 nAb titres after ZIKV infection were, however, higher than those induced in DENV-2 only infected animals suggesting that ZIKV induced low titres of cross-nAb against DENV. Surprisingly, DENV-2 infection of animals previously infected with ZIKV was not accompanied by an anamnestic increase in cross-nAb titres till about 1 week after DENV-2 infection. This delay coincided with enhanced DENV-2 viremia indicating that high levels of cross-bAb in the absence of high nAb contributes to enhancement of DENV infection. Serum collected 8 weeks after DENV-2 infection had high levels of nAb and showed delayed antibody dependent enhancement (ADE) of infection (1:100 dilution) as compared with serum that was collected from ZIKV infected animals prior to DENV-2 infection (1:10 dilution). Examination of serum from macaques that were simultaneously infected with both ZIKV and DENV-2 showed high levels of nAb and delayed ADE responses raising the possibility that the low levels of cross-nAb induced by ZIKV infection could be overcome by co-immunization against ZIKV and DENV infection. Taken together, our results provide additional insights into the nature and kinetics of cross-reactive antibody responses and identify a critical correlate that could potentially prevent enhancement of DENV infection during ZIKV convalescence.

## Introduction

Zika virus (ZIKV) is a flavivirus that exhibits high levels of serological cross-reactivity with dengue virus (DENV)^1–5^. We recently reported that prior exposure to ZIKV significantly enhances DENV infection in rhesus macaques in vivo^6^. Serum from ZIKV immune animals displayed a significant capacity to mediate in vitro antibody dependent enhancement (ADE) of all four serotypes of DENV suggesting a potential role for cross-reactive antibodies induced by ZIKV in ADE of DENV infection. ADE has been well documented in DENV infections^7–12^ and others have reported that ZIKV-induced antibodies enhance DENV-2 replication in vitro^13^. Previous studies have shown that serum from both acute and convalescent DENV-infected subjects demonstrate significant neutralizing antibody (nAb) activity against ZIKV suggesting that prior DENV infection could potentially prevent infection with ZIKV^3^. On the other hand, Montoya et al.^14^ examined antibody cross-neutralization after ZIKV and DENV infection and reported low levels of cross-reactivity in ZIKV-infected subjects against DENV-1 to 4 serotypes. Likewise, other studies have reported that ZIKV induces little or no cross-nAb responses against DENV^15,16^.

In line with the above studies, rhesus macaques previously infected with ZIKV were shown to have PRNT_50_ titres of <1:10 against DENV at the time of DENV challenge, though these animals had significant levels of DENV cross-reactive binding antibodies (bAb)^6^. This finding raised the possibility that conformational DENV cross-reactive epitopes capable of inducing nAb responses against DENV were likely minimally immunogenic thereby contributing to low levels of cross-neutralizing activity after DENV infection. We sought to address this question using serum that was collected longitudinally from ZIKV convalescent macaques after infection with DENV-2. We hypothesized that if ZIKV infection induced DENV specific nAb responses then we would detect an anamnestic amplification of these responses immediately after DENV-2 challenge. We examined sera from ZIKV convalescent animals at 0, 1, 4, 7, and 56 days after DENV-2 infection and compared these responses to DENV-2 only infected animals. Our results show that ZIKV infection induces DENV cross-nAb but at titres that remain below the detectable levels (<1:10) during the first few days after DENV-2 infection when there was significant enhancement of viremia. Anamnestic increases in cross-nAb titres become readily detectable (>1:10) by day 7 post-DENV-2 infection that coincided with a decrease in DENV-2 viremia.

## Results

### Kinetics of cross-reactive IgM and IgG responses induced by Zika and Dengue virus

**Fig. 1:**
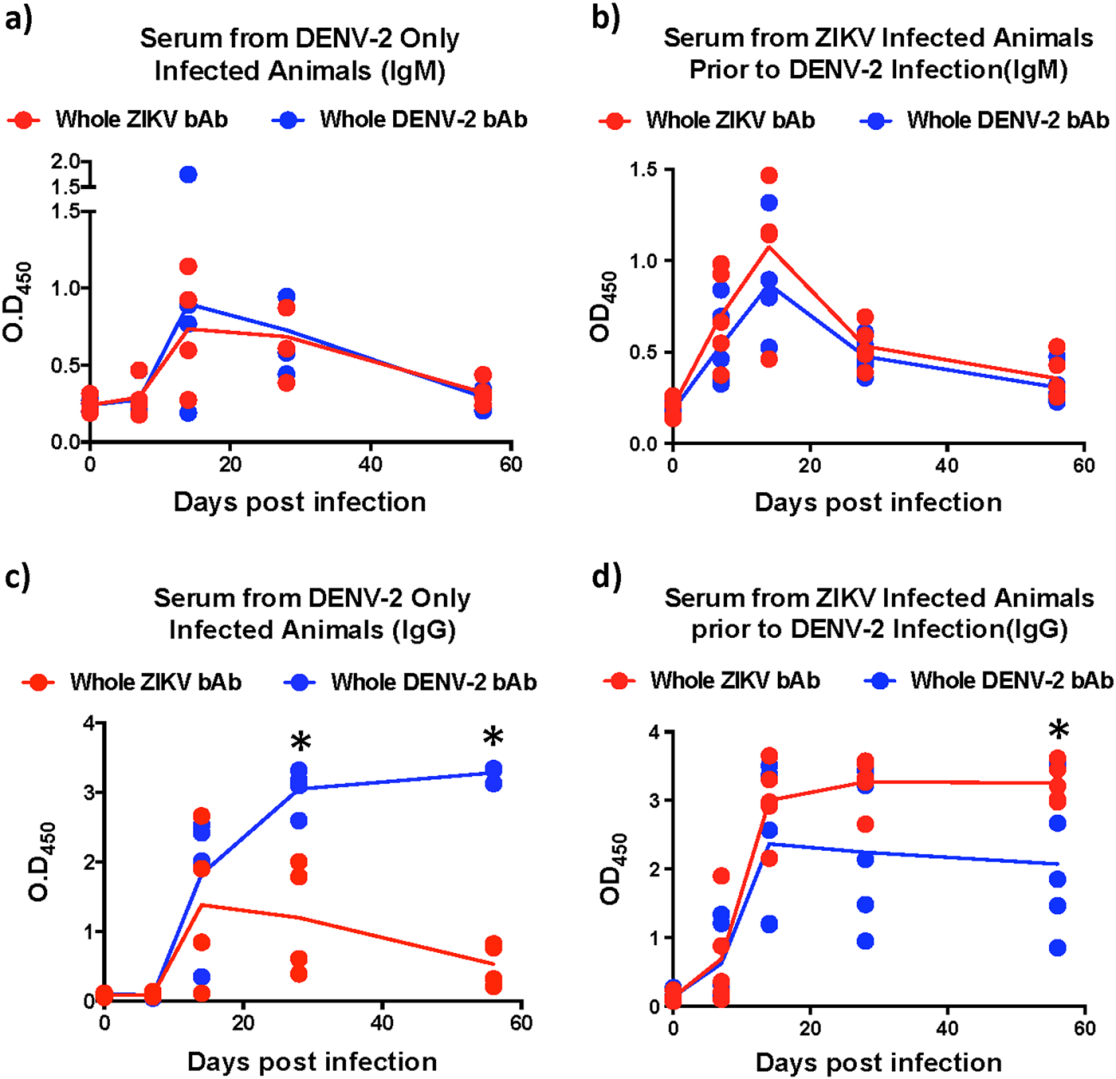
Kinetics of cross-reactive antibody responses. Relative binding antibody (bAb) levels against whole ZIKV and DENV-2 were examined using ELISA. The kinetics of binding IgM in serum that was collected longitudinally from (**a**) DENV-2 only (ZIKV naïve) infected animals (*n* = 4), (**b**) ZIKV-infected animals prior to DENV-2 infection (*n* = 5). The kinetics of binding IgG in serum that was collected longitudinally from (**c**) DENV-2 only (ZIKV naïve) infected animals (*n* = 4), (**d**) ZIKV-infected animals prior to DENV-2 infection (*n* = 5). Statistical significance was determined using multiple unpaired *t*-tests and corrected for multiple comparisons using the Holm-Sidak method. A *p* *<* 0.05 was considered significant and * indicates significant difference between groups

Numerous studies have reported induction of cross-reactive antibodies by related Flaviviruses. There is, however, limited information regarding the early kinetics of responses induced by ZIKV and DENV. To address this gap we examined the evolution of DENV-2 cross-reactive IgM and IgG responses induced by ZIKV infection prior to DENV-2 infection and compared them to DENV only infected animals (Fig. 1). Our results show that similar levels of cross-reactive IgM responses were induced against ZIKV and DENV in both groups of animals that peaked at day 14 after infection (Fig. 1a–b). Interestingly, IgM levels induced in DENV-2 only infected animals were less apparent at day 7 PI than those induced in ZIKV-infected animals prior to DENV-2 infection. IgM responses declined to near pre-infection levels by 56 days postinfection (PI).

Unlike IgM responses, we observed interesting differences in the kinetics of IgG levels between the two groups of animals (Fig. 1c–d). In DENV-2 only infected animals, both DENV-2 and ZIKV binding IgG levels remained near baseline at day 7 PI and become discernable by day 14 PI (Fig. 1c). DENV-2 binding IgG levels peaked at day 28 PI whereas, ZIKV binding IgG peaked earlier at day 14 PI. Unlike DENV-2 binding IgG that remained high during the first 56 days of infection, ZIKV binding IgG levels declined significantly to near baseline levels by 56 days PI suggesting a divergence in the kinetics of cross-reactive IgG responses induced by DENV-2 infection in ZIKV naïve animals (Fig. 1c).

In contrast to DENV-2 only infected animals, the levels of both DENV-2 and ZIKV binding IgG in ZIKV-infected animals were readily detectable by day 7 PI (Fig. 1d). Binding IgG levels peaked at day 14 and stayed high during the first 56 days of infection. As expected, the levels of ZIKV binding IgG were significantly higher than DENV-2 binding IgG at day 56 PI (Fig. 1d).

### Both ZIKV and DENV induce little or no cross-neutralizing antibody responses

**Fig. 2:**
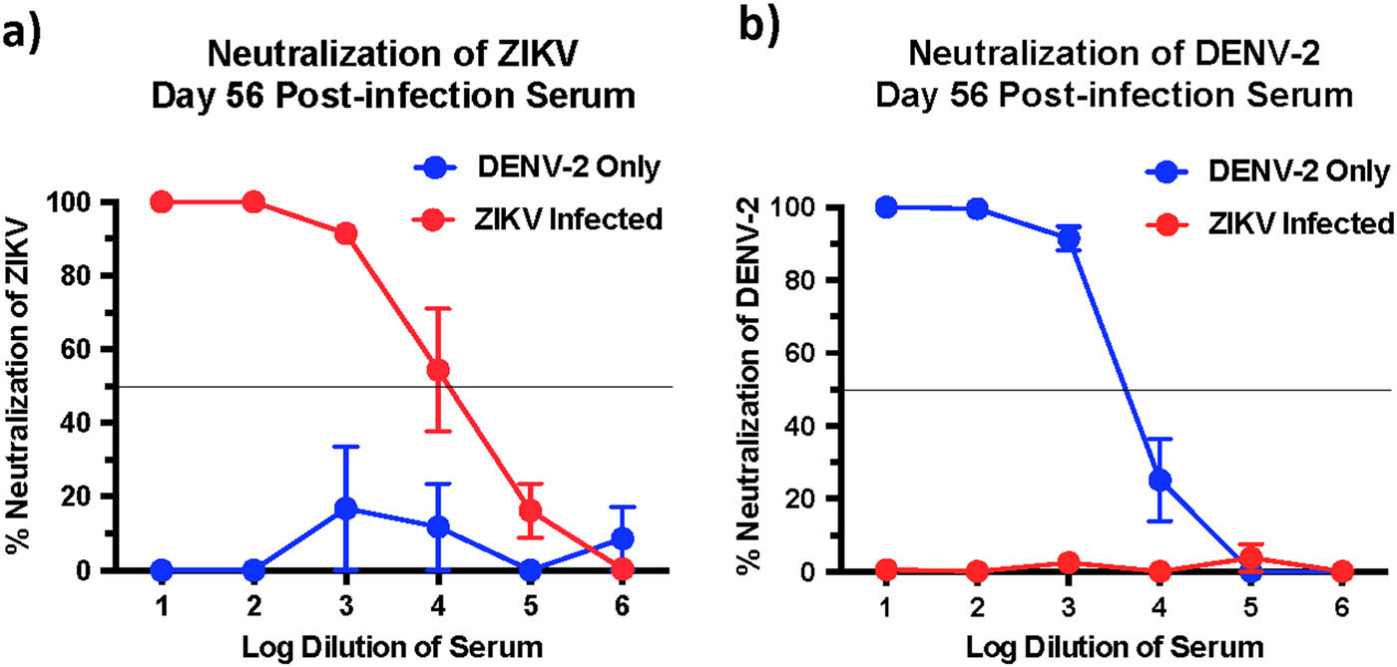
ZIKV-infected animals display little or no cross-neutralizing antibody responses against DENV-2. Percentage neutralization of (**a**) ZIKV and (**b**) DENV-2 by serum that was collected longitudinally from DENV-2 only (ZIKV naïve) (*n* = 4) and ZIKV-infected animals prior to DENV-2 infection (*n* = 5). Line represents PRNT_50_ titres

Next we determined if the high levels of cross-reactive bAb responses induced during both ZIKV and DENV-2 infection was accompanied by the induction of cross-nAb responses against both these viruses. To address this question we first examined the ZIKV and DENV-2 nAb titres in serum that was collected at 56 days PI using plaque reduction neutralizing test (PRNT). We observed significantly high titres of ZIKV nAb in ZIKV-infected animals, and high DENV-2 nAb titres in DENV-2 only infected animals (Fig. 2a–b); both groups displayed a PRNT_50_ titre of 1000–10,000.

DENV-2 only infected animals induced little or no cross-nAb responses against ZIKV with a PRNT titre of <1:10 (Fig. 2a). Likewise, the PRNT_50_ titres against DENV-2 in ZIKV-infected animals were <1:10 (Fig. 2b) suggesting that infection with either ZIKV or DENV-2 alone induces little or no cross-nAb activity in vivo. These findings were not surprising, as previous studies have reported that serum from ZIKV-infected human subjects failed to cross-neutralize DENV^17^. Likewise Dejnirattasai et al.^18^ showed that plasma from DENV-infected donors variably neutralized different strains of ZIKV.

**Fig. 3:**
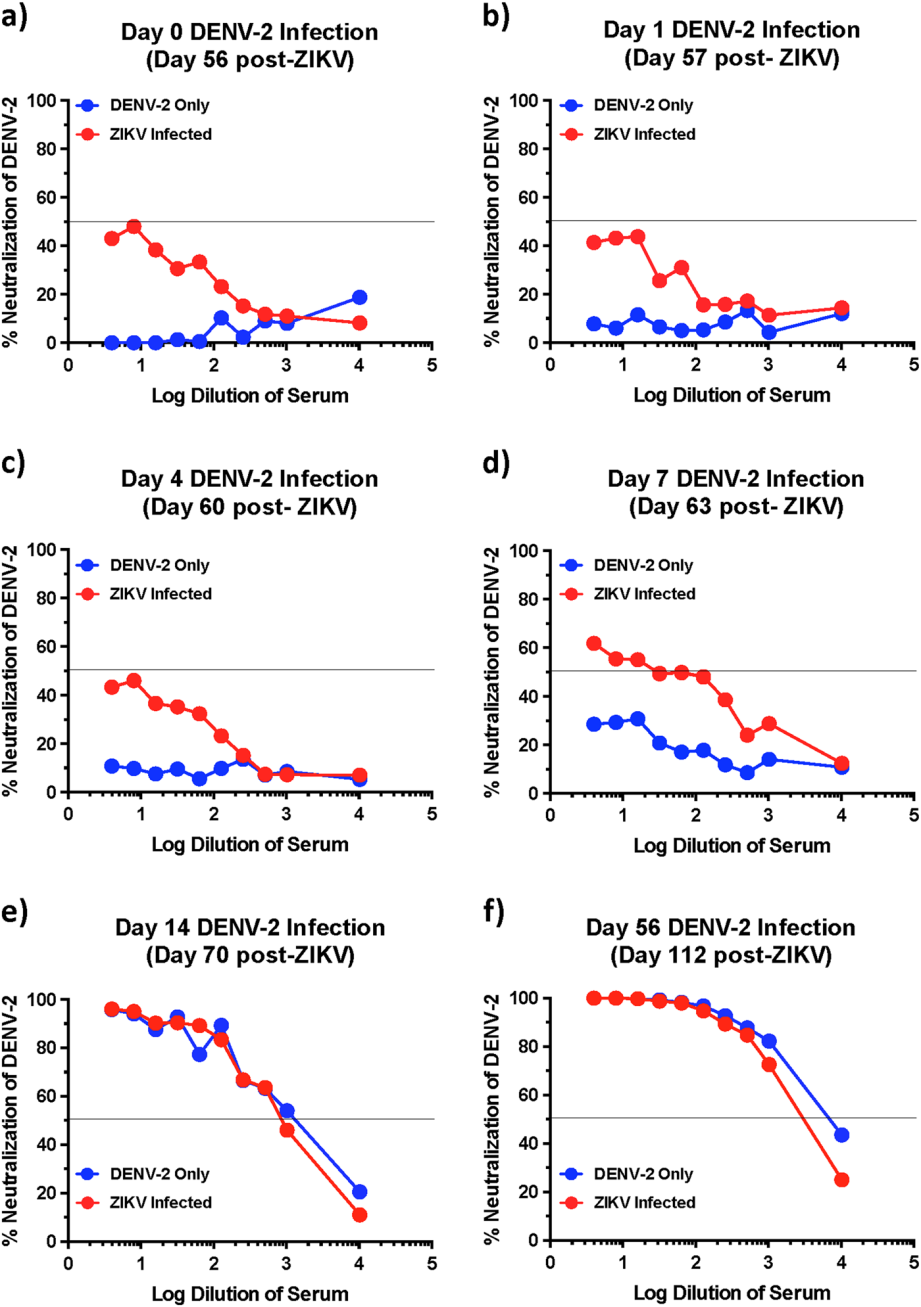
ZIKV immune animals display delayed anamnestic cross-neutralizing antibody responses after DENV-2 infection. Kinetics of neutralizing antibody responses against DENV-2 using serum that was collected longitudinally from DENV-2 only (ZIKV naïve) and ZIKV-infected animals at (**a**) day 0, (**b**) day 1, (**c**) day 4, (**d**) day 7, (**e**) day 14, and (**f**) day 56 after DENV-2 infection (*n* = 5). Line represents 50% neutralization

To confirm if nAb activity against DENV-2 was completely absent in macaques infected with ZIKV and not neutralizing at lower dilutions, we examined nAb titres against DENV-2 using 2-fold dilution of serum that was collected at 56 days after ZIKV infection (Fig. 3a); we observed low levels of neutralization against DENV-2 in ZIKV-infected animals prior to DENV-2 infection (day 0 of DENV-2 challenge; ~40% neutralization) as compared with DENV-2 only infected animals (day 0 of DENV-2 challenge; ~10% neutralization) suggesting that prior infection with ZIKV-induced DENV cross-nAb responses albeit at significantly low levels. These results appear to be in line with studies suggesting that ZIKV lies outside the DENV serocomplex^14^.

### Anamnestic neutralizing antibody responses remain low during the first few days after Dengue infection in ZIKV infected animals

Previous studies have shown the titres of DENV specific nAb correlate with reduced probability of severe DENV infection^19^. To determine if low nAb played a role in the enhancement of DENV-2 infection in ZIKV-exposed animals, we examined the early kinetics of DENV-2 nAb responses in serum that was collected from ZIKV-infected animals at days 1, 4, 7, 14, and 56 after DENV-2 infection and compared them to day 0 of DENV-2 infection (day 56 post-ZIKV infection). Neutralizing antibody titres during the first 4 days after DENV-2 infection did not differ from that of day 0 levels suggesting that there was no anamnestic increase in the nAb titres immediately after infection (Fig. 3a–c). Neutralizing antibody titres increased by day 7 post-DENV-2 infection to detectable levels with titres <1:100 (Fig. 3d). Detectable nAb titres at day 7 post-DENV-2 infection coincided with a decline in plasma viremia (Table 1). By day 14 and 56 post-DENV-2 infection, nAb responses against DENV-2 were readily detectable (Fig. 3e–f). These findings indicate that DENV-2 infection failed to boost cross-nAb induced by prior ZIKV infection during the early stages of infection.

**Table 1:** Kinetics of DENV-2 viremia

	Log plasma viral loads ± SD	
Days post-infection	DENV-2 only infected animals	ZIKV-infected animals
1	1.7 ± 0.0	1.7 ± 0.0
2	3.1 ± 1.0	1.8 ± 0.2
3	3.1 ± 1.0	4.0 ± 0.7
4	3.5 ± 1.2	5.4 ± 0.4
5	4.7 ± 0.6	6.5 ± 0.6
7	4.4 ± 0.3	3.5 ± 1.3
14	2.3 ± 1.1	1.7 ± 0.0
28	1.7 ± 0.0	1.7 ± 0.0
56	1.7 ± 0.0	1.7 ± 0.0

(*SD*) Standard deviation

**Fig. 4:**
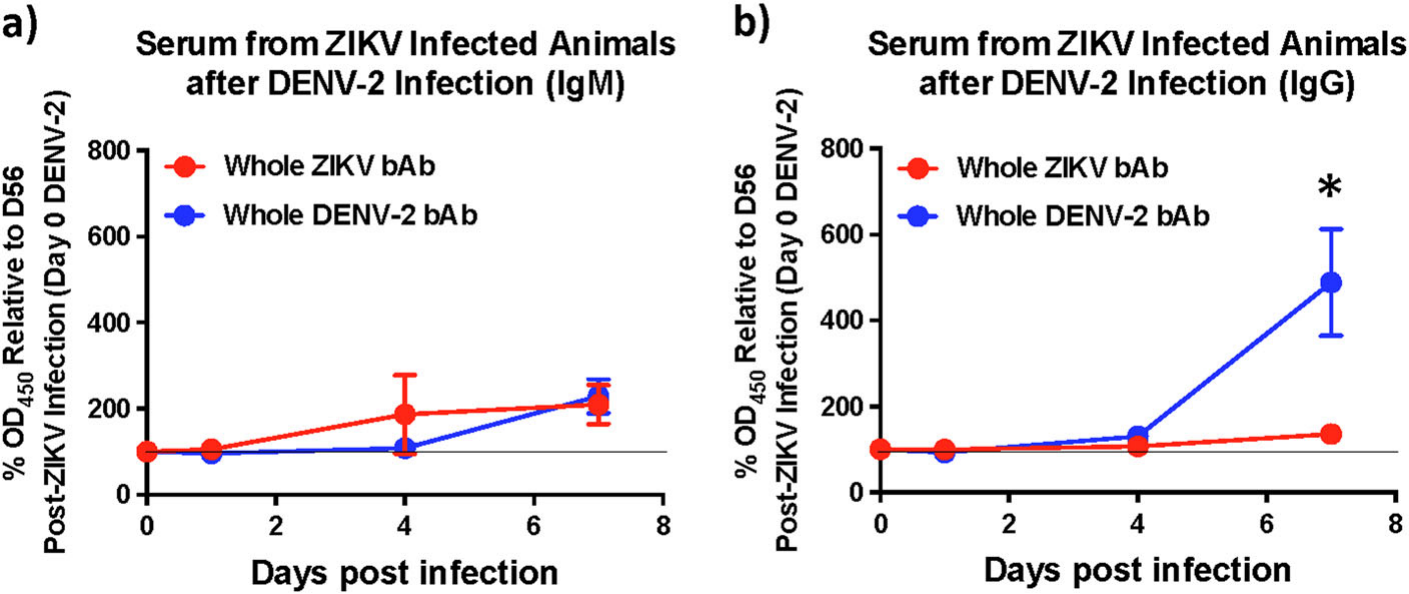
ZIKV infected animals display delayed binding antibody responses after DENV-2 infection. Kinetics of binding antibody (bAb) responses against DENV-2 in serum showing relative levels of (**a**) IgM and (**b**) IgG against DENV-2 using serum that was collected longitudinally from ZIKV-infected animals 1, 4, and 7 days after DENV-2 infection (*n* = 5). Percentage OD_450_ values relative to each animals day 56 post-ZIKV infection (day 0 DENV-2) values are shown. Line represents day 0 vales. Statistical significance was determined using multiple unpaired *t*-tests and corrected for multiple comparisons using the Holm-Sidak method. A *p* *<* 0.05 was considered significant and * indicates significant difference between groups

As enhancement of DENV infection has been associated with binding cross-reactive antibodies in the absence of detectable nAb activity, we assessed the levels of envelope binding IgM and IgG at 1, 4, and 7 days after DENV-2 infection using whole virus-based ELISA assays to determine if bAb levels unlike nAb were boosted immediately after infection (Fig. 4a–b). Similar to nAb titres, we observed little or no anamnestic increase in either DENV-2 binding IgM or IgG levels during the first 4 days after infection with DENV-2. However, by day 7 post-DENV-2 infection the levels of cross-reactive IgG had increased significantly suggesting that there was an anamnestic antibody response that became apparent only 1 week after infection. These findings suggest that the enhancement of DENV-2 viremia likely occurred due to the high prevalence of cross-reactive binding IgG that were induced by ZIKV infection prior to DENV-2 infection and remained high at the time of DENV-2 infection. It is difficult to directly discern from our studies if the higher binding IgG responses we observed 7 days after DENV-2 infection was an anamnestic or a naïve response to DENV-2 challenge. However, based on the kinetics of IgG responses in DENV-2 only infected animals where IgG levels were near baseline levels at day 7 post-DENV-2 infection (Fig. 1c), it is possible to infer that the enhanced IgG response at day 7 post-DENV-2 in ZIKV-exposed animals were most likely consisted of an anamnestic IgG response.

### ZIKV infection induces predominantly IgG1 subclass of ZIKV specific and DENV cross-reactive IgG

**Fig. 5:**
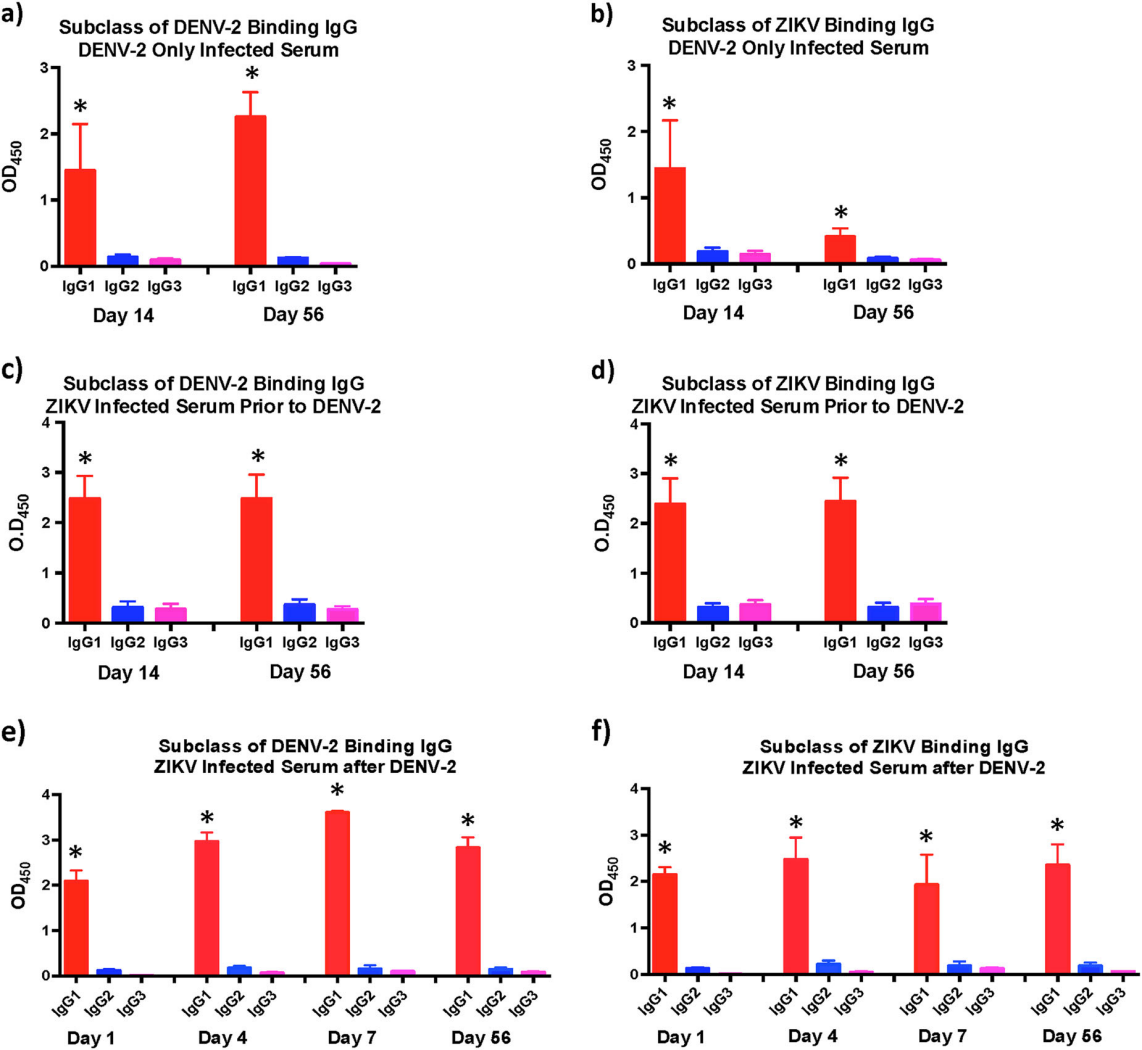
IgG1 is the primary subclass of IgG in serum from ZIKV naïve and infected animals prior to and after infection with DENV-2. Relative levels of (**a**) DENV-2 and (**b**) ZIKV binding IgG1, IgG2, and IgG3 in serum that was collected at day 14 and day 56 post-infection from DENV-2 only (*n* = 4) infected animals. Relative levels of (**c**) DENV-2 and (**d**) ZIKV binding IgG1, IgG2, and IgG3 in serum that was collected at day 14 and day 56 post-infection from ZIKV-infected animals prior to DENV-2 infection (*n* = 5). Relative levels of (**e**) DENV-2 and (**f**) ZIKV binding IgG1, IgG2, and IgG3 in serum that was collected at day 1, 4, 7, and 56 post-infection from ZIKV-infected animals after DENV-2 infection (*n* = 5). Statistical significance was determined using one-way ANOVA and differences between time-points were determined by post-hoc analysis using Tukey’s multiple comparisons test. A *p* *<* 0.05 was considered significant. Error bars represent standard error

We have previously shown that DENV-2 enhancement of viremia in ZIKV infected  animals was accompanied by release of various pro-inflammatory mediators associated with severe DENV infections^6^, and others^12^ have implicated IgG1 subclass of antibodies in the induction of dengue hemorrhagic fever (DHF)/dengue shock syndrome (DSS). To determine if ZIKV infection induced different subclasses of IgG, we examined the ZIKV and DENV-2 envelope binding IgG1, 2, and 3 in serum from ZIKV infected animals that was collected at day 14 and 56 PI prior to DENV-2 infection using whole virus based ELISA and compared them to DENV-2 only infected animals (Fig. 5a–d). We could not get reliable reagents to IgG4 subclass hence restricted our analysis to the three subclasses. Positive controls were setup simultaneously for each subclass using purified rhesus macaque IgG1, 2, and 3 (Supplementary Fig. 1).

Our results showed that the predominant subclass of IgG induced against ZIKV and DENV-2 in DENV only infected animals were IgG1 (Fig. 5a–b). Likewise, both DENV-2 and ZIKV cross-reactive IgG subclass induced in ZIKV-infected animals were IgG1 (Fig. 5c–d). There was no major change in the subclass of IgG in both groups of animals between day 14 and 56 PI. DENV-2 infection in ZIKV-infected animals did not alter the subclass of IgG with the primary subclass of IgG induced against both ZIKV and DENV-2 being IgG1 (Fig. 5e–f).

### The potential for in vitro ADE decreases with higher neutralizing antibody titres

**Fig. 6:**
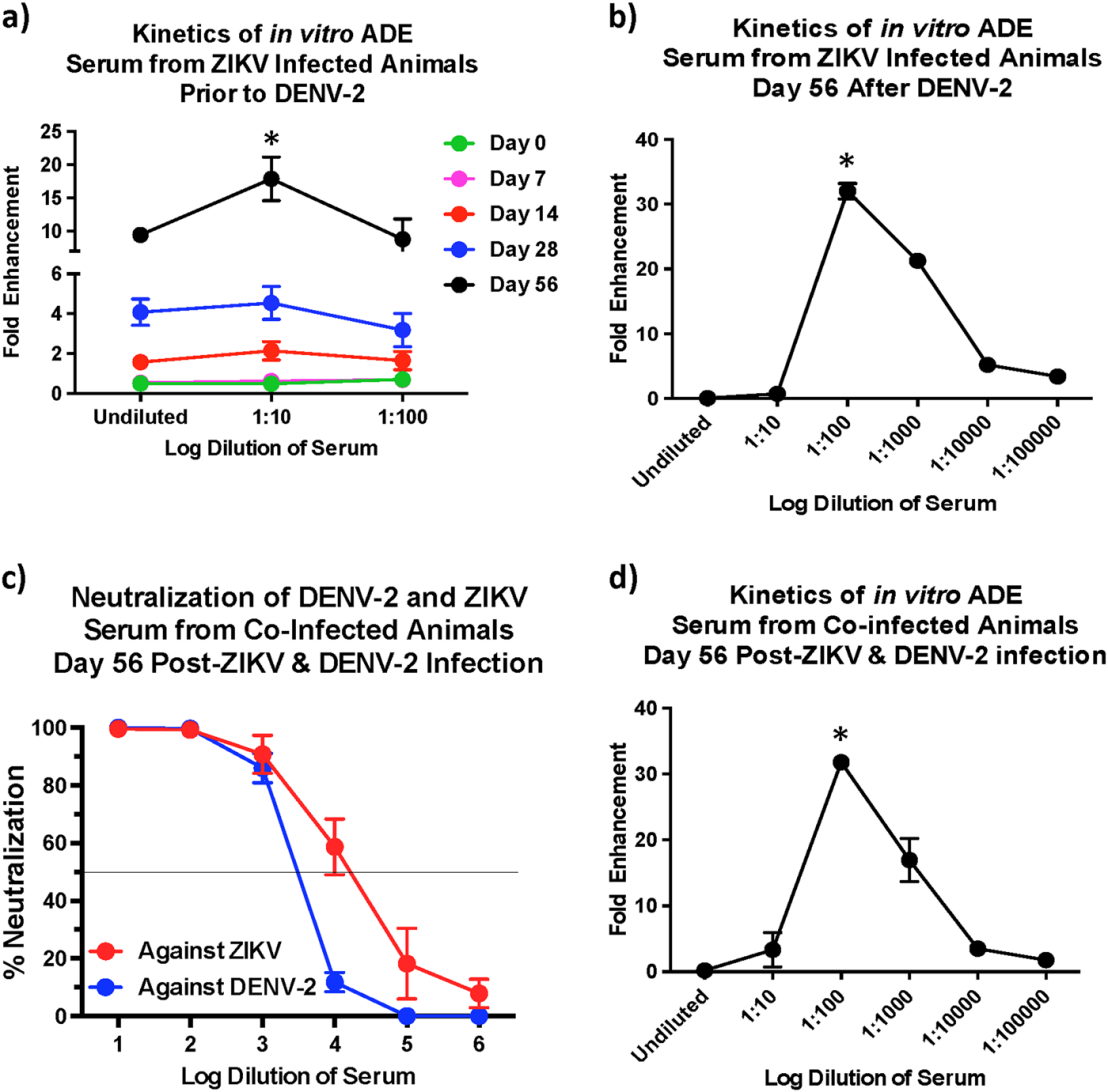
Serum from ZIKV infected animals collected after infection with DENV-2 demonstrate antibody dependent enhancement at a dilution of 1:100. Fold enhancement of DENV-2 infection in K562 cells using serum that was collected (**a**) from ZIKV-infected animals (*n* = 5) prior to DENV-2 infection and (**b**) at day 56 after DENV-2 infection. Statistical significance was determined using One-way ANOVA and differences between time-points were determined by post-hoc analysis using Tukey**’**s multiple comparisons test. A *p* < 0.05 was considered significant. (**c**) Percentage neutralization of DENV-2 using serum that was collected at day 56 post-infection from animals that was simultaneously infected with ZIKV and DENV-2 (*n* = 5). (**d**) Fold enhancement of DENV-2 infection in K562 cells using serum that was collected at 56 days from rhesus macaques that were simultaneously infected with ZIKV and DENV-2 (*n* = 5). Statistical significance was determined using One-way ANOVA and differences between time-points were determined by post-hoc analysis using Tukey's multiple comparisons test. A *p* < 0.05 was considered significant. Error bars represent standard error

Our earlier studies^6^ have shown that serum from ZIKV-exposed animals prior to DENV-2 infection displayed a significant potential for ADE that peaked at a serum dilution of 1:10. To assess if the in vitro ADE of DENV-2 infection was altered in the presence higher DENV-2 nAb titres, we compared the potential for in vitro ADE using sera from the ZIKV-infected animals that was collected prior to DENV-2 infection (Fig. 6a) and at 56 days after DENV-2 infection (Fig. 6b). Our results showed that enhancement peaked at a dilution of 1:100 at day 56 post-DENV-2 infection as compared with a 1:10 dilution in serum that was collected at day 56 after ZIKV infection but prior to DENV-2 challenge suggesting that the threshold for enhancement is significantly higher in the presence of elevated nAb titers against DENV-2.

### Simultaneous infection with ZIKV and DENV-2 induces high neutralizing antibody titres against both viruses

Given the correlation between high nAb responses and low in vitro ADE, we hypothesized that higher nAb levels if induced simultaneously against both ZIKV and DENV-2 could likely reduce the potential for enhancement of DENV infection. We tested this hypothesis by co-infecting rhesus macaques with ZIKV and DENV-2 simultaneously and assessing nAb titres (Fig. 6c), and the potential for in vitro ADE of DENV-2 infection (Fig. 6d). Our results showed that coinfection induced high ZIKV and DENV-2 nAb titres that were similar to the nAb titres induced against DENV-2 in DENV-2 only infected animals or against ZIKV in ZIKV exposed animals prior to DENV-2 infection (Fig. 2a–b). Sera from the coinfected animals that were collected at 56 days post-infection displayed in vitro ADE of DENV-2 infection at a dilution of 1:100 (Fig. 6d) as compared with a 1:10 dilution for sera that was collected prior to DENV-2 infection (Fig. 6a) suggesting that higher nAb titres delays the induction of ADE responses in vitro.

## Discussion

Antibody cross-reactivity among related flaviviruses have been extensively documented. Less clear is the effectiveness of cross-nAb responses in protection from infection. Though enhancement of infection with heterologus serotypes of DENV has been well studied, there is limited information regarding the potential for ZIKV-induced responses to protect from DENV infection. We have previously reported enhancement of DENV viremia in rhesus macaques that had been previously exposed to ZIKV. Here we show that this enhancement occurred in the presence of high titres of pre-existing cross-reactive bAb with little or no DENV cross-nAb activity. Previous studies^16^ have shown that ZIKV infection induced little or no cross-reactive nAb against DENV. Likewise Dick et al.^15^ using intracerebral neutralization test in mice showed that ZIKV immune serum failed to protect mice from DENV challenge.

What was surprising, however, was the lack of an early anamnestic antibody response in ZIKV infected animals immediately after DENV infection, a time period that coincided with enhancement of viremia. ZIKV infected animals displayed significant enhancement of DENV-2 viremia as compared with ZIKV naïve animals that peaked at 5 days post-DENV-2 infection and, declined by day 7 postinfection (Table 1). These findings were somewhat surprising given the high levels of DENV-2 viremia in ZIKV infected animals during the first week of infection; plasma DENV-2 viral loads were ~ 4 logs/ml of plasma at day 3, ~5.4 logs/ml at day 4, and ~6.5 logs/ml at day 5. The lack of significant anamnestic boosting of ZIKV-induced DENV cross-nAb responses likely points to the time it takes for a secondary immune response to emerge. On the other hand, enhancement was likely dictated by the high levels of pre-existing cross-reactive bAb that were present at the time of DENV-2 infection.

We examined enhancement in ZIKV-infected animals during early convalescence (56 days post-ZIKV infection). Would cross-reactive titres remain at such high levels over a longer period of convalescence, and if those levels can enhance in vivo infection is not known and still remains to be determined. We have previously shown that serum that was collected from macaques 16 weeks after ZIKV infection enhanced infection in vitro with all serotypes of DENV^29^. Additional studies are needed to examine the effect of sera from long-term convalescent subjects to determine if the potential for enhancement exists over long periods of time. It was, however, exciting to note that the decline in enhanced DENV viremia in ZIKV immune animals coincided with the emergence of detectable nAb responses against DENV raising the possibility that high cross-neutralizing antibody titres if induced could potentially prevent the enhancement of DENV infection even in the presence of high levels of binding cross-reactive antibody responses. In line with this argument, the in vitro capacity of serum to induce ADE was significantly higher very early after DENV-2 infection as compared with serum that was collected at 8 weeks post-DENV-2 infection when nAb titres against DENV-2 was significantly high; peak enhancement was observed at a dilution of 1:10 using sera that was collected prior to DENV-2 infection as compared with 1:100 dilution of serum that was collected 56 days after DENV-2 infection. It is interesting to note the nAb titres though below detectable levels in ZIKV infected animals prior to DENV-2 infection (<1:10 at day 0), were higher than those induced in ZIKV naïve DENV-2-infected animals during the first 1 week after infection (Fig. 3a–d); ~10% neutralization in ZIKV naïve DENV-2-infected animals vs. ~40% neutralization at day 56 post-ZIKV infection prior to DENV-2 infection suggesting that there are shared conformational nAb inducing epitopes between ZIKV and DENV that could potentially be harnessed for protection. Robbiani et al.^20^ reported that antibody responses induced against DENV-1 envelope domain III prior to ZIKV infection was associated with higher ZIKV specific nAb titers after ZIKV infection. These antibodies appeared to cross-neutralize DENV-1 and ZIKV but not other flaviviruses. Valiant et al.^21^ recently reported that serum from a human subject with high levels of nAb against both ZIKV and DENV displayed in vitro ADE of DENV infection at a dilution of 1:10,000 as compared with a 1:10 dilution of serum from a subject who had high levels of nAb only against ZIKV but not against DENV. Could these responses translate to protection from enhancement in vivo is not clear and remains to be determined.

Vaccine-based strategies using a combination of ZIKV and DENV vaccines could potentially induce such high levels of nAb responses against both ZIKV and DENV. Though we did not directly address this hypothesis using challenge studies, we did observe significantly high nAb titres against both ZIKV and DENV-2 in rhesus macaques that were coinfected with ZIKV and DENV-2 at the same time suggesting that vaccinating against both viruses simultaneously could likely protect from enhancement. Additional studies are needed to address this hypothesis.

It was interesting to note that the primary subclass of IgG induced after ZIKV infection against both whole ZIKV and DENV viruses was IgG1. The subclass did not change after DENV-2 infection of ZIKV infected animals. The exact reasons for preferential induction of IgG1 is not clear and may be related to the specific microenvironment induced within the B cell follicles. The induction of IgG1, however, may have implications for the enhancement of DENV infection as numerous studies have reported. Wang et al.^12^ demonstrated that patients with DHF/DSS had cross-reactive non-neutralizing antibodies that had a high affinity for FcγRIIIA and were primarily of IgG1 subclass. Thein et al.^22^ showed that serum from subjects with acute DHF or DSS contained higher levels of anti-DENV IgG1 subclass of IgG. Likewise, Posadas-Mondragon et al.^23^ reported that IgG1 subclass of DENV specific IgG was the predominant subclass during both primary and secondary DENV infections that significantly increased in the patients with secondary DENV infection and DHF. Others^24^ have shown that high levels of DENV specific IgA, IgG1, and IgG4 antibodies in serum were markers of high risk for DSS and DHF.

In conclusion, our findings provide additional new insights into the early kinetics of cross-bAb and nAb responses induced against DENV following ZIKV infection and raise the prospect that higher levels of nAb if induced against both viruses simultaneously could potentially overcome the effect of enhancing bAb and protect from adverse events such as enhancement of DENV infection during ZIKV convalescence.

## Materials and methods

### Samples

Archived serum samples collected from rhesus macaques were used in this study. All animal experiments were reviewed and approved by Institutional Animal Care and Use Committee at Bioqual Inc., and samples were acquired through a tissue sharing protocol. ZIKV naïve animals were infected with 10^5^ TCID_50_ of DENV-2 (strain 16681) subcutaneously for a period of 8 weeks and serum samples were collected longitudinally at day 0, 7, 14, 28, and 56 post-DENV-2 infection. Likewise serum was collected longitudinally at day 0, 7, 14, 28, and 56 from animals who had been infected subcutaneously with 10^6^ TCID_50_ of ZIKV (Puerto Rico Strain; Genbank KU501215) for a period of 8 weeks (*n* = 5). Additionally, serum samples were obtained after challenge with 10^5^ TCID_50_ DENV-2 (strain 16681) subcutaneously at day 0 (day 56 post-ZIKV infection), 1 (day 57), 4 (day 61), 7 (day 63), 14 (day 70), and 56 (day 112) post-DENV-2 infection (*n* = 5). Plasma viral loads were determined as described previously^6^. To assess the effect of co-infection, we collected serum at day 56 postinfection from rhesus macaques that were simultaneously infected with ZIKV and DENV-2 as described above (*n* = 5). Samples from each animal were assayed separately for each of the time points examined, and in all of the assays reported in the manuscript.

### Plaque reduction neutralization test (PRNT)

Fully confluent monolayer of Vero cells were infected with ZIKV or DENV-2 in DMEM containing 1% FBS. When severe cytopathic effect was observed the supernatant was collected, centrifuged at 500*g* for 10 min, and stored at −80 °C. The titer of the stocks was determined by plaque assay using Vero cells.

PRNT assays were set up in 24-well plates that were set to 100% confluence with Vero cells. Serum was heat inactivated for 30 min at 56 °C. Serum was initially diluted at 1:5 in DMEM with 1% FBS followed by a 10 × serial dilution. ZIKV or DENV-2 were diluted in DMEM containing 1% FBS to a titer of ~4 × 10^2^ plaque forming units (PFU)/ml. An equal volume of virus was added to each dilution of serum, mixed, and incubated for 1 h at 37 °C. After aspirating the media from the 24-well plate with Vero cells, 75 uL of the virus and serum mix was added to each well. Each dilution was assayed in duplicate. After incubating the plates at 37 °C in 5% CO_2_ for 1 h, 1 ml of 2 × EMEM containing 10% FBS that was mixed with an equal volume of 2% methylcellulose, warmed to 37 °C, was added to each well. The plates were incubated for 5 days at 37 °C in 5% CO_2_. Plates were fixed with 10% formalin, stained with hematoxylin and eosin, and the number of plaques in each well was counted. Percentage neutralization was determined using the formula: (number of plaques in dilution of interest)/(number of plaques in virus only control well) × 100. The operator was blinded to the identity of the samples prior to each assay.

### Neutralization assay using RVP

Neutralization assays using DENV reporter viral particles (RVP) were performed as described previously^6,25^. Briefly, serum was heat inactivated for 30 min at 56 °C and diluted 2-fold in RPMI-10. The diluted serum was mixed in equal volume with 5 uL of DENV-2 RVP (Integral Molecular, Philadelphia, PA) and incubated at 37 °C in 5% CO_2_ for one hour. After incubation, 40,000 Raji DC-SIGN-R cells^26^ (NIH AIDS Reagent Program, Division of AIDS, NIAID, NIH: Raji/DC-SIGN Cells from Drs. Li Wu and Vineet N. KewalRamani) were suspended in 15 μl of RPMI-10 and added to the serum/RVP mixture and incubated at 37 °C in 5% CO_2_ for one hour. Following incubation 100 uL of RPMI was added and the cells were incubated at 37 °C in 5% CO_2_ for 3 days in a 96 well U-bottom tissue culture plate. After culture cells were fixed in 0.5% Paraformaldehyde, and analyzed using an LSR II flow cytometer. Each sample was assayed in duplicate. Collected data was analyzed using Flowjo 9.6 software. The operator was blinded to the identity of the samples prior to each assay and the data was unblinded after analysis.

### ELISA for serum IgM and IgG

 96-well plates were coated overnight at 4 °C with a pan-flavivirus antibody (clone 4G2), followed by blocking with 1% BSA in 1xPBS overnight at 4 °C. The 4G2 antibody has been extensively used in capture-based ELISA to look at both ZIKV and DENV binding antibody levels in serum^3,18,27,28^. After washing with PBS-T (1xPBS with 0.5% Tween-20) cell culture stocks of ZIKV or DENV-2 diluted 1:10 in 1xPBS (~1–2 × 10^4^ pfu/ml) were added to each well and the plate was incubated for 60 min at 37 °C. Serum samples were diluted 1:100 in PBS-T and dispensed into respective wells. PBS-T alone was used as reagent blanks. After incubating for 60 min at 37 °C, the plates were washed 3 × with PBS-T, and anti-IgG-HRP (Biorad) diluted 1:50,000 in PBS-T or anti-IgM-HRP (Rockland), diluted 1:10,000 was added to each well. The plates were incubated for 30 min at room temperature, washed 3× with wash buffer and Tetramethylbenzidine (TMB) substrate was added to each well. The reaction was stopped after 15 min using 2 M Sulfuric acid, and the OD was determined at 450 nm using an ELISA plate reader. Samples were assayed in duplicate and the operator was blinded to the identity of the samples prior to each assay and data was unblinded after analysis.

### IgG subclass ELISA

Subclass of serum IgG was determined as described previously^29^. Cell culture stocks of ZIKV or DENV-2 were diluted 2:5 in PBS and bound to half area 96-well plate overnight at 4 °C, followed by blocking with 1% BSA in PBS overnight at 4 °C. Serum samples were diluted 1:10 in PBS-T and incubated on the virus coated plates for 60 min at 37 °C. Mouse anti-rhesus macaque IgG1, IgG2, and IgG3 were obtained from the NIH Nonhuman Primate Reagent Resource supported by AI126683 and OD010976, and added to each well at a dilution of 1:200 in PBS-T. The plates were incubated for 60 min at 37 °C. After washing with PBS-T goat anti-mouse HRP was added at a dilution of 1:10,000 in PBS-T for 30 min at room temperature. The plate was washed and TMB solution was added to each well. The reaction was stopped after 15 min using 2M Sulfuric acid and the OD was determined at 450 nm using an ELISA plate reader. Purified rhesus macaque IgG1, 2, and 3 that was obtained from the NIH Nonhuman Primate Reagent Resource were used a positive controls (Supplementary Fig. 1). Samples were assayed in duplicate and the operator was blinded to the identity of the samples prior to each assay and data was unblinded after analysis

### Antibody dependent enhancement assay

The capacity of serum to enhance DENV infection in vitro was performed using RVP as described previously^6,25^. Serum was heat inactivated at 56 °C for 30 min, serially diluted 10 fold, and mixed with 5 μL of RVP at a ratio of 1:1 volume. After incubation for 1 h at 37 °C in 5% CO_2_, the serum/RVP mix was added to 40,000 K562 cells^30^ suspended in 15 μl of RPMI-10 and incubated for 1 h at 37 °C in 5% CO_2_. After 1 h the cells were washed twice with RMPI-10 to remove unbound virus, resuspended in RPMI-10 and cultured in a 96 well tissue culture plate for 48 h. After culture cells were fixed in 0.5% Paraformaldehyde, and analyzed using an LSR II flow cytometer. Collected data was analyzed using Flowjo 9.6 software. Samples were assayed in duplicate and the operator was blinded to the identity of the samples prior to each assay and data was unblinded after analysis.

### Data analysis

Statistical analysis was performed using GraphPad Prism Version 5.0 software (GraphPad Prism Software, Inc., San Diego, CA). Error bars represent standard error and *p* < 0.05 was considered significant.

## Electronic supplementary material

Supplementary Figure 1
