# Supplementary figures and images for: Zika convalescent macaques display delayed induction of anamnestic cross-neutralizing antibody responses after dengue infection
Source: Emerg Microbes Infect. 2018 Jul 13;7:130. doi: 10.1038/s41426-018-0132-z (PMC6045599; doi:10.1038/s41426-018-0132-z)

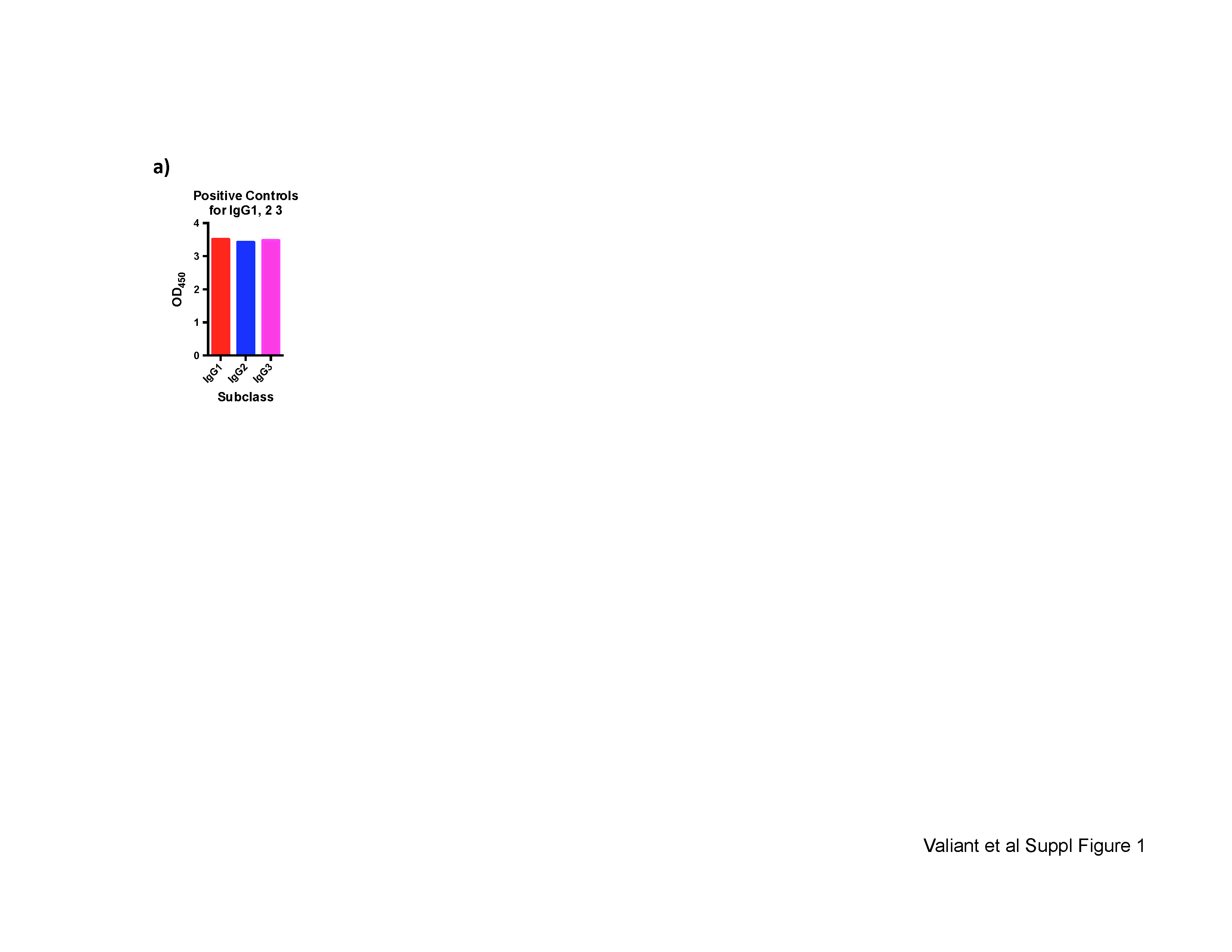

Supplement: Supplementary file 1 — Supplementary Figure 1 [file 41426_2018_132_MOESM1_ESM.tif]
